# Role of Dlg5/lp-dlg, a Membrane-Associated Guanylate Kinase Family Protein, in Epithelial-Mesenchymal Transition in LLc-PK1 Renal Epithelial Cells

**DOI:** 10.1371/journal.pone.0035519

**Published:** 2012-04-23

**Authors:** Takuhito Sezaki, Kohki Inada, Takayuki Sogabe, Kumiyo Kakuda, Lucia Tomiyama, Yohsuke Matsuno, Takafumi Ichikawa, Michinori Matsuo, Kazumitsu Ueda, Noriyuki Kioka

**Affiliations:** 1 Division of Applied Life Sciences, Graduate School of Agriculture, Kyoto University, Sakyo, Kyoto, Japan; 2 Institute for Integrated Cell-Material Sciences (iCeMS), Kyoto University, Sakyo, Kyoto, Japan; Northwestern University Feinberg School of Medicine, United States of America

## Abstract

Discs large homolog 5 (Dlg5) is a member of the membrane-associated guanylate kinase adaptor family of proteins, some of which are involved in the regulation of epithelial-to-mesenchymal transition (EMT). Dlg5 has been described as a susceptibility gene for Crohn's disease; however, the physiological function of Dlg5 is unknown. We show here that transforming growth factor-β (TGF-β)-induced EMT suppresses Dlg5 expression in LLc-PK1 cells. Depletion of Dlg5 expression by knockdown promoted the expression of the mesenchymal marker proteins, fibronectin and α-smooth muscle actin, and suppressed the expression of E-cadherin. In addition, activation of JNK and p38, which are stimulated by TGF-β, was enhanced by Dlg5 depletion. Furthermore, inhibition of the TGF-β receptor suppressed the effects of Dlg5 depletion. These observations suggest that Dlg5 is involved in the regulation of TGF-βreceptor-dependent signals and EMT.

## Introduction

Discs large homolog 5 (Dlg5)/placenta-dlg(p-dlg)/large type of p-dlg(lp-dlg)/KIAA0583 (hereafter referred to as Dlg5) is a member of the membrane-associated guanylate kinase (MAGUK) adaptor family of proteins, in which some members are involved in the regulation of EMT [1], [2]. The MAGUK family of proteins shares at least one PDZ (PSD-95, Dlg, ZO-1) domain, an SH3 domain, and a guanylate kinase domain. Dlg5 was initially proposed to be one of five human homologs of the *Drosophila* Discs large (Dlg) protein, a tumor suppressor gene in imaginal discs, based on similarities in sequence and domain structure [3]. However, recent computational analysis has revealed that Dlg5 does not belong to the DLG MAGUK subfamily defined by DLG1-4 in humans but rather has its own ortholog in other animals including *Drosophila* [4].

Dlg5 has been described as a susceptibility gene for Crohn's disease, one of two subphenotypes of inflammatory bowel disease [5], although the involvement of this gene in this disease is still controversial [6], [7]. Recent reports have shown that polymorphisms in Dlg5 are associated with Crohn's disease in a gender- and age-specific manner [8], [9], [10]. To examine whether Dlg5 plays a role in Crohn's disease and how Dlg5 affects the disease, the physiological function of Dlg5 should be clarified. We have isolated Dlg5 as a binding protein for vinexin, a protein localized to cell-cell adhesion sites and focal adhesions [11], [12]. We have also shown that Dlg5 associates with β-catenin and localizes to cell-cell adhesion sites [11]. Others have identified several other Dlg5 binding proteins [3], [13], [14]. Although reports have demonstrated that the Dlg5 gene knockout shows defects in the kidney and brain, the physiological function of Dlg5 in renal as well as intestinal epithelial cells remains poorly understood.

Epithelial cells can be converted into mesenchymal cells by a process called epithelial-to-mesenchymal transition (EMT) [15], [16], [17]. Epithelial cells form cell sheets that have strong cell-cell adhesions mediated by specialized structures, such as tight junctions, adherens junctions, and desmosomes. Epithelial cells show limited motile activity and have apical-basolateral polarity. Mesenchymal cells, in contrast, have higher motile activity, an elongated fibroblastic morphology, and anterior-posterior polarity. The process of EMT involves the disruption of tight cell-cell adhesions, loss of apical-basolateral polarity, and an acquisition of a higher motile activity, concomitant with a shift in cytoskeletal dynamics. This process is also associated with dramatic changes in gene expression, including a loss of junction components, such as E-cadherin, and an increase in mesenchymal markers, including fibronectin and α-smooth muscle actin (SMA) [17], [18]. Although it is well established that EMT plays an important role in various physiological and pathophysiological conditions, including embryogenesis, tumor invasion, and renal fibrosis [15], [16], [19], [20], [21], [22], the mechanisms regulating EMT are not fully understood.

Here we investigated the role of Dlg5 in the renal epithelial cell line, LLc-PK1, which exhibits high levels of Dlg5 expression. We found that transforming growth factor-β (TGF-β)-induced EMT suppressed the expression of Dlg5 in LLc-PK1 cells. Depletion of Dlg5 expression by RNAi-mediated knockdown promoted the expression of the mesenchymal marker proteins, fibronectin and SMA, and suppressed the expression of E-cadherin. Re-expression of Dlg5 attenuated the induction of SMA expression observed in Dlg5-depleted cells. Moreover, the induction of SMA expression by Dlg5 depletion was dependent on TGF-β receptor (TβR)-mediated signaling. These observations suggest that Dlg5 plays a role in TGF-β receptor-dependent signaling and EMT in LLc-PK1 cells.

## Methods

### Antibodies and Reagents

SP600125 and SB203580 were from Tocris Bioscience (Ellisville, MO). ALK5 inhibitor II was from WAKO Pure Chemical Industries (Osaka, Japan). TGF-β was obtained from R&D Systems (Minneapolis, MN). EZ-Link™ Sulfo-NHS-Biotin and monomeric-avidin conjugated agarose beads were purchased from Pierce.

The following antibodies were used in this study: anti-fibronectin (BD Transduction), anti-E-cadherin (BD Transduction), anti-β-tubulin (Sigma), anti-GFP (Santa Cruz Biotechnology), anti-SMA (Sigma), anti-β-catenin (BD Transduction), anti-p-Smad2 (Cell Signaling Technology), anti-Smad2 (Cell Signaling Technology), anti-p-JNK (Cell Signaling Technology), anti-JNK1 (Santa Cruz Biotechnology), anti-p38 (Cell Signaling Technology), anti-p-p38 (Cell Signaling Technology), and anti-vinculin (Sigma). The anti-Dlg5 and anti-vinexin antibodies were described previously [11], [23].

### Cell culture, plasmids, and transfection

Porcine renal epithelial LLc-PK1 cells (American Type Culture Collection) and PC3 cells (American Type Culture Collection) were maintained in Medium199 and RPMI1640 containing 10% fetal bovine serum, respectively. Experiments were performed on collagen IV coated dishes unless otherwise indicated. In some experiments, cells were incubated with TGF-β (4 ng/ml) for the indicated number of hours. FLAG-tagged Dlg5 has been described previously [11]. For lentivirus-mediated transfection, GFP-tagged Dlg5 was subcloned into pCDH-EF1-IRES-blasticidin (System Biosciences) and Dlg5 shRNA plasmid was purchased from Open Biosystems. Plasmids encoding the Smad7 and TGF-β receptors were gifts from Dr. S. Aota (RIKEN, Japan). Dominant negative mutant of JNK [24] and p38 [25] were kind gifts from Dr. S. Tamura (Tohoku University, Japan) and Dr. Y. Gotoh (University of Tokyo, Japan), respectively. Transfection of plasmid DNA was performed using Lipofectamine LTX (Invitrogen) or FuGENE HD (Roche Diagnostics K.) according to the manufacturer's instructions.

### RNA interference (RNAi)

The stealth siRNAs for Dlg5/lp-dlg (siDlg5#1, AAGCUCAGAAUAAGCGGAACUUGAU; siDlg5#2, CCAACCUUUCUUGUAGCCAGCUGAU) and for β-catenin (UAGAAGCUGGUGGAAUGCAAGCUUU) were designed based on the sequence of the porcine genome or porcine cDNA (FP085540, NM_214367) and synthesized by Invitrogen. The siRNA for GFP (GGGCACAAGCUGGAGUACAACUACA), which was used as a control, and the negative control siRNA3 were from Invitrogen. The transfection of siRNAs (10 nM) was performed using LipofectAmine RNAiMax (Invitrogen) as described [26].

### mRNA Quantification by Real-time PCR

Real-time PCR was performed as described [27]. Briefly, total RNA was isolated using an RNeasy Mini Kit (Qiagen). cDNA was generated from 100 ng of each total RNA sample using the Superscript III First-Strand Synthesis system (Invitrogen). The resulting cDNA samples were subjected to quantitative real-time PCR reactions using the StepOne Real-Time PCR System (Applied Biosystems). Independent PCRs were performed using the same cDNA for both the gene of interest (*Dlg5*, *E-cadherin*, and *snail*) and *GAPDH*, using the Fast SYBR Green Master Mix (Applied Biosystems) with the following gene-specific primer pairs: *Dlg5* forward, 5′-CTTCAAGCAACTTGCAGTTCAAG-3′, and reverse, 5′-TCAGAACCCACGACAGATCGT-3′; *E-cadherin* forward, 5′-CTCTGGCCGAAGCAGGATT-3′, and reverse, 5′-GCAAGGATGCCTCCCAGAAT-3′; *snail* forward, 5′- CTCGCAAGGCCTTCAACTG-3′, and reverse, 5′-GAGAGCACCCAGGCTGACA-3′; *GAPDH* forward, 5′-CCTCCTGTACCACCAACTGCTT-3′, and reverse, 5′-GCCGAAGTTGTCATGGATGA-3′. The primers were designed using Primer Express software (Applied Biosystems). A dissociation curve was generated at the end of each PCR cycle to verify that a single product was amplified. Each RNA sample was amplified in duplicate. The results were normalized to *GAPDH* mRNA as an internal control. The relative level of each mRNA was calculated by the “comparative CT method” in StepOne v1.0 software (Applied Biosystems). The values represent the mean ± S.D. from three independent experiments.

### Immunofluorescence microscopy

Cells were cultured on glass coverslips or, in some cases, on collagen IV-coated coverslips. The cells were fixed with methanol for 15 minutes at −20°C for Dlg5 immunostaining or with 4% paraformaldehyde containing 5% sucrose for 30 minutes at room temperature and permeabilized in 0.2% TritonX-100/PBS for 5 minutes. They were then blocked with 10% goat serum/PBS (+) for 1 hour and incubated with primary antibodies for 1 hour. The cells were stained with Alexa Fluor-labeled secondary antibody (Molecular Probes) for 45 minutes. Fluorescence images were taken with a BX51 microscope (Olympus) equipped with an ORCA-ER CCD camera (Hamamatsu Photonics, Hamamatsu, Japan), a PASCAL confocal microscopy system (Carl Zeiss Co., Ltd), or a LSM700 confocal microscopy system (Carl Zeiss Co., Ltd).

### Detection of cell surface proteins

LLc-PK1 cells transfected with control or Dlg5 siRNA were washed with ice-cold PBS and incubated with 0.5 mg/ml sulfo-NHS-biotin (Pierce) in PBS for 30 min on ice in the dark. The cells were washed with PBS to remove unbound sulfo-NHS-biotin and lysed in RIPA buffer containing protease- and phosphatase- inhibitors as previously described [28]. Equal amounts of protein were incubated with monomeric-avidin conjugated agarose beads (Pierce) for 2 hours at 4°C to precipitate the biotinylated proteins. The precipitated proteins were subjected to SDS-PAGE and detected by immunoblotting.

### Statistical analysis

Statistical analysis was performed using Student's paired *t*-test.

## Results

### TGF-β disrupts the epithelial cell morphology of LLc-PK1 cells and decreases Dlg5 expression

**Figure 1:**
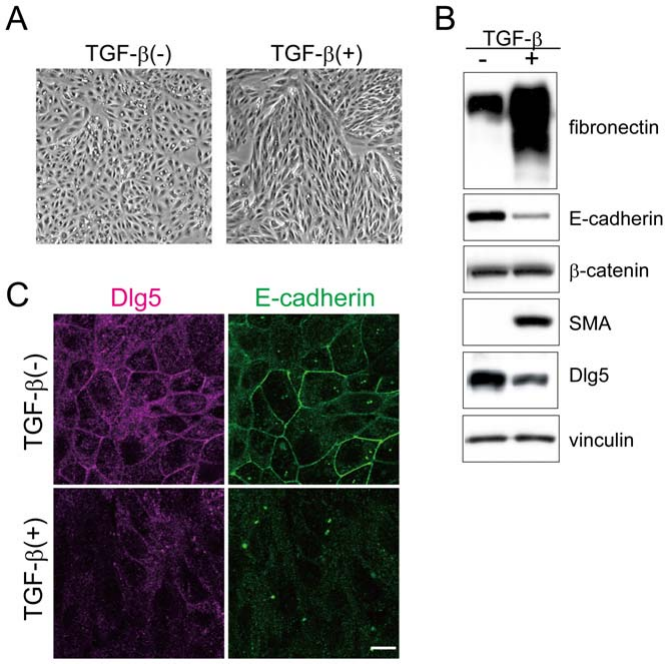
TGF-β-mediated EMT decreases Dlg5 expression. **A:** LLc-PK1 cells were incubated with 4 ng/ml of TGF-β for three days. Images were taken using phase contrast microscopy. TGF-β treatment induced morphological changes of LLc-PK1 cells. **B:** Three days after incubation with TGF-β, cells were lysed and immunoblotted using the indicated antibodies, which include an antibody for the epithelial marker E-cadherin, antibodies for the mesenchymal markers SMA and fibronectin, and antibodies for Dlg5 and β-catenin. As a loading control, vinculin expression was detected. **C:** LLc-PK1 cells were incubated with 4 ng/ml of TGF-β for three days. The cells were immunostained with anti-Dlg5 or anti-E-cadherin antibody. The scale bar indicates 10 µm. TGF-β treatment induced EMT and decreased Dlg5 expression. The results are representative of at least three independent experiments.

Some members of the MAGUK family of proteins, including Dlg1 and ZO-1, are involved in EMT [1], [2]. Thus, we first examined whether TGF-β-induced EMT affects Dlg5 expression. We used renal proximal tubular epithelial LLc-PK1 cells because Dlg5 is highly expressed in these cells as well as in kidney of humans and mice [11], [13]. LLc-PK1 cells were incubated with TGF-β for three days. Treatment with TGF-β changed the cell morphology from a typical epithelial cell-like to an elongated fibroblast like-morphology (Fig. 1A). Compared with control cells, the expression of E-cadherin (an epithelial marker) was lower, and the expression of SMA and fibronectin (mesenchymal markers) was higher in TGF-β-treated cells (Fig. 1B), indicating that TGF-β stimulation induced EMT in LLc-PK1 cells as previously reported [29], [30]. As shown in Fig. 1B, TGF-β-induced EMT downregulated the expression of Dlg5 in LLc-PK1 cells. Real-time PCR analysis showed that Dlg5 mRNA was decreased in TGF-β-treated cells (Fig. S1A). Interestingly, the expression of both vinexin α and β, which associate and colocalize with Dlg5 at cell-cell adhesion sites, was also decreased in TGF-β treated cells, although the expression of vinculin was not affected (Fig. S1B). Although N-cadherin expression is increased by EMT in other cells [17], [18], a significant increase in N-cadherin expression was not detected in LLc-PK1 cells under the conditions examined (data not shown). Immunofluorescence analysis also showed that Dlg5 expression was downregulated in TGF-β-treated cells (Fig. 1C). Both Dlg5 and E-cadherin localized to cell-cell adhesion sites and co-localized in control cells, as we have previously reported [11]. TGF-β stimulation reduced the signals of Dlg5 and E-cadherin at adhesion sites. These observations suggest that Dlg5 may be involved in TGF-β-induced EMT or maintenance of epithelial cell morphology in LLc-PK1 cells.

### Decreasing Dlg5 expression disrupts epithelial cell morphology and induces the expression of mesenchymal marker proteins

**Figure 2:**
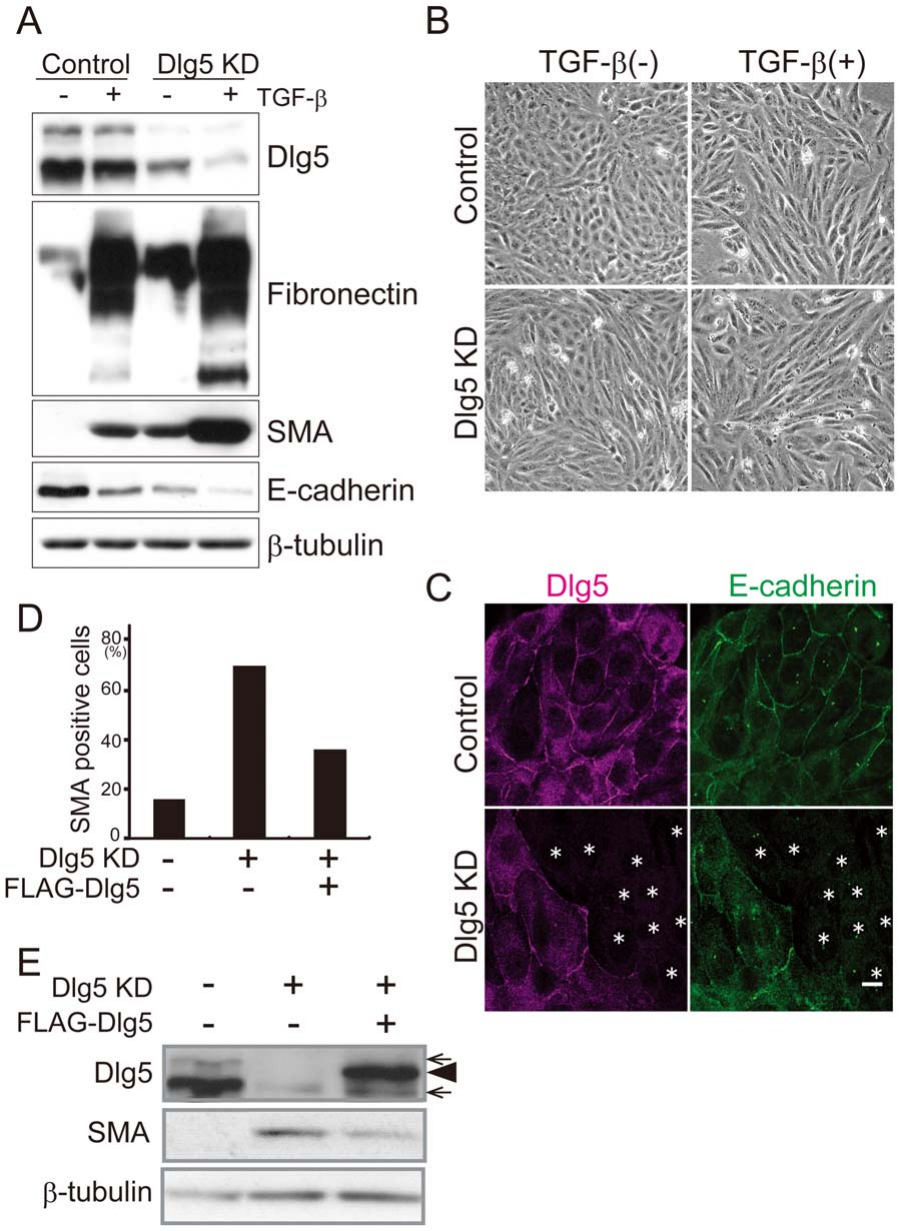
Dlg5 depletion disrupts epithelial cell morphology and induces the expression of mesenchymal marker proteins. **A:** LLc-PK1 cells were transfected with Dlg5 siRNA (siDlg5#1: KD) or control siRNA and then incubated with 4 ng/ml of TGF-β for three days. The cells were lysed and immunoblotted using the indicated antibodies. Expression of β-tubulin was examined as a loading control. The upper band observed in immunoblotting with anti-Dlg5 is the larger variant of Dlg5. **B:** Cells were treated as in A and incubated for 60 hours. The cells were then photographed using phase contrast microscopy to examine cell morphology. **C:** Cells were transfected with Dlg5 siRNA or control siRNA and then immunostained using anti-Dlg5 or anti-E-cadherin antibodies. The asterisks indicate the cells that were transfected with Dlg5 siRNA. The scale bar indicates 10 µm. **D:** LLc-PK1 cells were transfected with Dlg5 siRNA or control siRNA and incubated for 24 hours. A GFP expression plasmid was then transfected into the cells with FLAG-Dlg5 or control plasmids. After incubation for two days, the cells were immunostained using an anti-SMA antibody. Fifty GFP-expressing cells were randomly selected, and the fluorescent intensity of SMA staining was examined. The graph shows the ratio of cells with higher SMA expression than background. **E:** LLc-PK1 cells were transfected with FLAG-Dlg5 or control plasmid and incubated for six hours. The cells were further transfected with Dlg5 siRNA or control siRNA. After two days of incubation, the cells were lysed and immunoblotted using the indicated antibodies. Arrows indicate endogenously expressed Dlg5, and an arrow head indicates FLAG-Dlg5. Dlg5 depletion induced the expression of mesenchymal marker proteins, and the re-expression of Dlg5 suppressed it.

To investigate a potential role of Dlg5 in EMT, siRNA specific for Dlg5 was transfected into LLc-PK1 cells. Immunoblotting showed that cells transfected with the Dlg5-specific siRNA (siDlg5#1) exhibited reduced levels of Dlg5 by 80% compared to cells transfected with control siRNA (Fig. 2A). We found that the depletion of Dlg5 expression by knockdown disrupted the epithelial cell morphology of LLc-PK1 cells and that the cells developed an elongated fibroblast-like morphology (Fig. 2B). Furthermore, E-cadherin expression was downregulated, and the expression of SMA and fibronectin was upregulated in Dlg5-depleted cells (Fig. 2A). We also confirmed the decrease in E-cadherin protein expression by immunofluorescence analysis (Fig. 2C) and in E-cadherin mRNA levels by real-time PCR (Fig. S2). To further investigate the effect of Dlg5 depletion, another siRNA for Dlg5 (siDlg5#2) was transfected into LLc-PK1 cells. The reduced expression of Dlg5 was confirmed by immunoblotting. The siDlg5#2-induced reduction of Dlg5 expression also led to a decrease in E-cadherin expression and an increase in fibronectin and SMA expression (Fig. S3). However, the increase in SMA was smaller than that observed in siDlg5#1-transfected cells, possibly due to the cytotoxic effects of siDlg5#2. Because Dlg5 knockdown had the most prominent effect on SMA expression, we focused on SMA expression in the following experiments.

To corroborate the effect of Dlg5 depletion, we tested whether re-expression of Dlg5 suppresses the induction of SMA in Dlg5-depleted cells. Endogenously expressed porcine Dlg5 was reduced by the transfection of siRNA specific for porcine *Dlg5*. A plasmid encoding FLAG-tagged human Dlg5 or a control plasmid was then co-transfected with a GFP-expressing plasmid into LLc-PK1 cells. SMA expression in GFP-expressing cells was examined by immunofluorescence analysis. As shown in Fig. 2D, SMA expression was detected in only a small number of control cells (<20%). In contrast, 70% of Dlg5-depleted cells expressed SMA. As expected, re-introduction of human *Dlg5* into Dlg5-depleted cells decreased the number of SMA-expressing cells. Western blot analysis was also performed to investigate the effect of Dlg5 re-expression. Re-expression of FLAG-Dlg5 in Dgl5-depleted cells suppressed SMA expression (Fig. 2E). These results suggest that Dlg5 is crucial for the regulation of EMT or the maintenance of the epithelial features of LLc-PK1 cells.

To investigate whether Dlg5 function in the regulation of EMT is cell-type specific, we used PC3 cells, which are derived from human prostate cancer. Prostate is one of tissues expressing high levels of Dlg5 ([3] and unpublished results). Dlg5 expression in PC3 cells was knocked down by transfection of an shRNA plasmid for human Dlg5. Expression of Dlg5 was reduced by 90% in the shRNA-transfected cells. We found that E-cadherin expression was decreased, but fibronectin expression was increased in these cells (Fig. S4). This result suggests that the function of Dlg5 in EMT regulation is not specific for LLc-PK1 cells but can be observed in other cell types.

**Figure 3:**
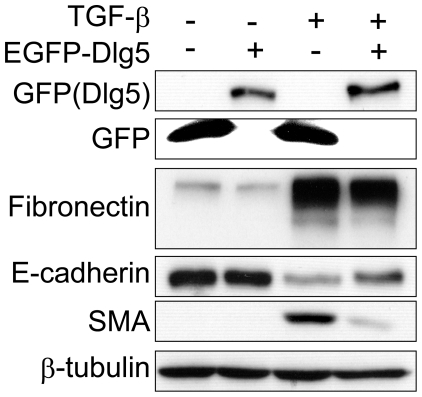
Overexpression of Dlg5 attenuates the TGF-β-mediated increase in the expression of mesenchymal marker proteins. LLc-PK1 cells stably expressing GFP or GFP-tagged Dlg5 were treated with or without 4 ng/ml TGF-β. After two days of incubation, cells were lysed and protein expression was detected by immunoblotting using the indicated antibodies. Exogenously expressed Dlg5 suppressed the increase in SMA and fibronectin expression.

We next examined whether Dlg5 overexpression inhibits EMT induced by TGF-β. LLc-PK1 cells overexpressing GFP-tagged Dlg5 were established by using lentivirus-mediated transfection. As shown in Fig. 3, Dlg5 overexpression suppressed TGF-β-induced fibronectin and SMA expression and slightly rescued E-cadherin expression. Together, these results suggest that Dlg5 is involved in EMT induced by TGF-β

### Dlg5 depletion promotes JNK and p38 activation

We next examined which molecules mediate the effect of Dlg5 depletion on EMT. β-catenin is one candidate because it enhances EMT in several cell systems [15], [17] and associates with Dlg5 physically [11], [13]. Dlg5 depletion, however, did not affect β-catenin expression or the subcellular distribution of β-catenin between the nucleus and the cytosol (data not shown). To further investigate the role of β-catenin in EMT induced by Dlg5 depletion, both Dlg5 and β-catenin were knocked down simultaneously in LLc-PK1 cells (Fig. S5). The expression of β-catenin was successfully reduced by 80% in the siRNA-transfected cells. However, knockdown of β-catenin did not suppress the expression of SMA or fibronectin induced by Dlg5 depletion. β-catenin knockdown also did not rescue E-cadherin expression, although E-cadherin expression was reduced regardless of TGF-β stimulation or Dlg5 depletion, possibly due to the destabilization of E-cadherin without β-catenin. Together, these observations suggest that molecules other than β-catenin are involved in the Dlg5-mediated regulation of EMT.

We next investigated Snail and Slug expression, EMT-regulating transcriptional factors. Although Western blotting using several commercially available antibodies did not detect either Snail or Slug protein expression (data not shown), real-time PCR analysis revealed a slight increase in the expression of *Snail* mRNA after Dlg5 depletion (Fig. S6). The increase was much less than that observed in TGF-β treated cells. *Slug* mRNA was hardly detected both in Dlg5-depleted and TGF-β treated cells. Together, these results suggest that Snail may have a role in the Dlg5-mediated regulation of EMT.

**Figure 4:**
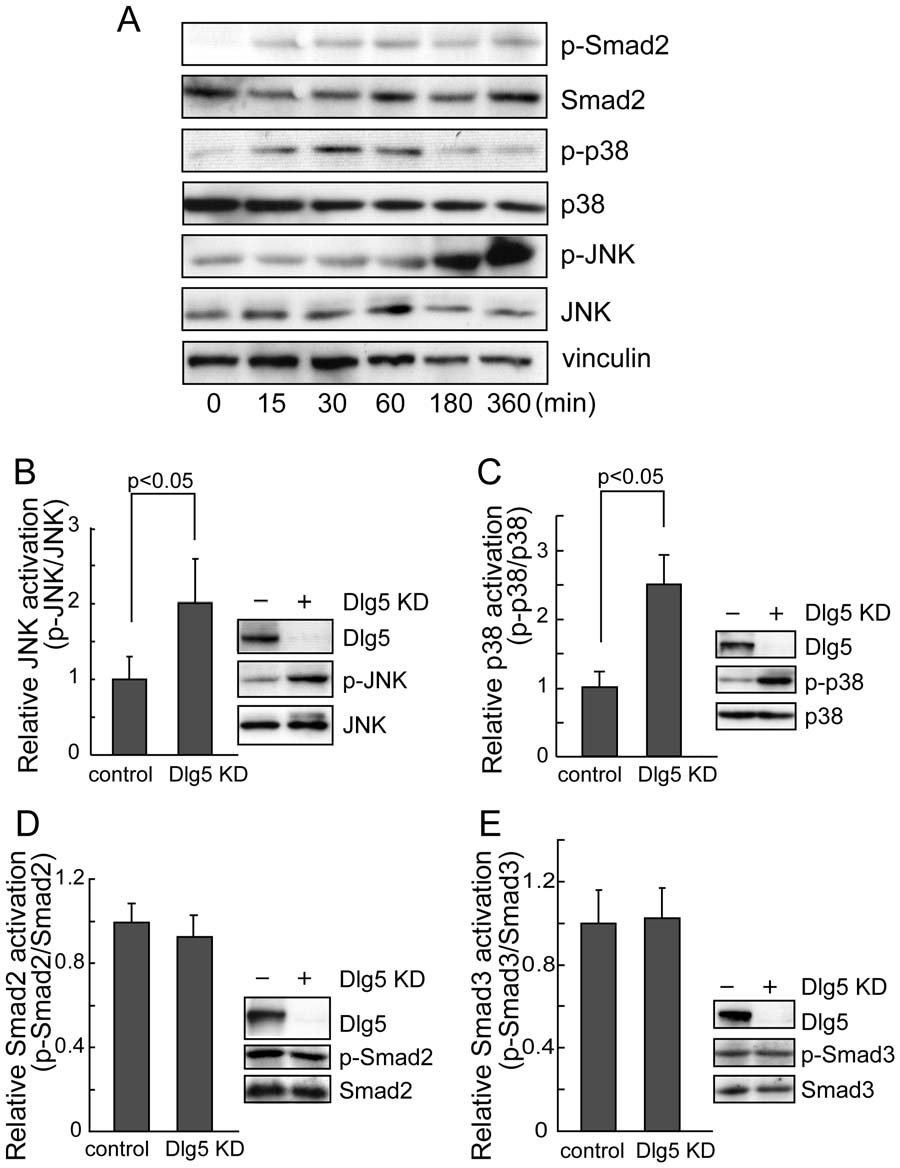
Dlg5 depletion promotes JNK and p38 activation. **A:** LLc-PK1 cells were stimulated with 4 ng/ml TGF-β and incubated for the indicated periods. Cells were then lysed and immunoblotted using the indicated antibodies. **B, C, D, E:** LLc-PK1 cells were transfected with Dlg5 siRNA (KD) or control siRNA and incubated for two days. Cell lysates were then immunoblotted using the indicated antibodies, and the results were quantitated. The values represent the mean ± S.E. from at least three independent experiments. Dlg5 depletion promoted JNK and p38 activation but not Smad2/3 activation.

Next, we focused on TGF-β-induced signaling. TGF-β stimulation increases the phosphorylation of regulatory Smads, such as Smad2 and Smad3. Once phosphorylated, these Smads form a complex with Smad4, which leads to their translocation into the nucleus and the transcriptional activation of various target genes. In addition, TGF-β activates the non-Smad pathway, which includes MAP kinase signals, such as JNK and p38 [31], [32]. Both Smad and non-Smad pathways stimulate EMT. In LLc-PK1 cells, TGF-β stimulation increased the phosphorylation of Smad2 as well as p38 and JNK MAP kinases (Fig. 4A). However, Smad3 phosphorylation was not increased in LLc-PK1 cells (data not shown). Thus, we investigated the effects of Dlg5 depletion on Smad and non-Smad signals (Fig. 4B–E). Two days after transfection of Dlg5 siRNA (siDlg5#1), a moderate but statistically significant (2-fold) increase in JNK activation was observed even without TGF-β stimulation (P<0.05). Dlg5-depleted cells also showed a 2.5-fold increase in p38 activation (P<0.05). Because these increases were detected two days after transfection of siRNA, these activations seem to be sustained. In contrast, TGF-β stimulation induces strong but only transient activation of these signals. Thus, moderate activation of JNK and p38 induced by Dlg5 knockdown seems to play an important role. Enhanced activation of JNK and p38 were also observed in siDlg5#2-transfected cells (data not shown). Dlg5 depletion slightly enhanced JNK and p38 activation even in TGF-β stimulated cells (data not shown), possibly because strong transient activation of JNK and p38 induced by TGF-β obscures the enhancement induced by Dlg5 depletion. In contrast, phosphorylation of Smad2 and Smad3 was not affected by Dlg5 depletion, either with (Fig. 4D, E) or without TGF-β stimulation (data not shown). These results suggest that Dlg5 attenuates the activation of JNK and p38 MAP kinases in LLc-PK1 cells.

### Inhibition of MAP kinases suppresses Dlg5 depletion-mediated EMT

**Figure 5:**
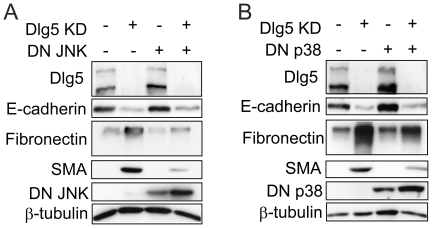
Dominant-negative mutants of JNK and p38 suppress the expression of mesenchymal marker proteins in Dlg5-depleted cells. **A:** Six hours after transfection of a dominant-negative JNK mutant or a control plasmid into LLc-PK1 cells, the cells were further transfected with control or Dlg5 siRNA. After incubation for two days, expression of the indicated proteins was investigated by immunoblotting. **B:** Dominant-negative p38 mutant or a control plasmid was transfected into LLc-PK1 cells and treated and analyzed as in A. Expression of β-tubulin was detected as a loading control. The results are representative of at least three independent experiments. The expression levels of dominant-negative mutants were affected by Dlg5 knockdown by an unknown mechanism. Dominant-negative mutants of JNK and p38 suppressed the expression of fibronectin and SMA.

To investigate whether these MAP kinase signals are involved in Dlg5 depletion-induced EMT, dominant-negative mutants of MAP kinases were used. LLc-PK1 cells were transfected with a dominant negative JNK mutant, followed by transfection of control or Dlg5 siRNA. As shown in Fig. 5A, expression of the dominant-negative JNK mutant clearly suppressed the increases in fibronectin and SMA expression induced by Dlg5 depletion and slightly rescued E-cadherin expression. Considering the transfection efficiency of the plasmid (∼40%), dominant-negative JNK suppressed most increases in fibronectin and SMA expression and moderately rescued E-cadherin. Relatively small effects on E-cadherin expression may indicate that other signals are also involved in E-cadherin expression. Inhibition of JNK activity by an inhibitor (SP600125) also suppressed the increase in fibronectin expression induced by Dlg5 depletion (data not shown). Together, these observations indicate the importance of JNK activation in Dlg5 depletion-induced EMT in LLc-PK1 cells.

We also examined the effect of inhibiting p38 kinase. LLc-PK1 cells were first transfected with a dominant-negative p38 mutant followed by the transfection of siRNA. Expression of the dominant-negative p38 mutant exhibited effects on fibronectin, SMA, and E-cadherin expression as the expression of the dominant-negative JNK mutant (Fig. 5B). Inhibition of p38 kinase activity by an inhibitor (SB203580) also suppressed the increase in fibronectin expression induced by Dlg5 depletion (data not shown). All together, these results suggest that both JNK and p38 kinase signaling induced by Dlg5 depletion are involved in the regulation of EMT.

### EMT induced by Dlg5 depletion involves TGF-β receptor-mediated signaling

TGF-β stimulation transmits signals *via* two serine-threonine kinase receptors, type I (TβRI) and type II (TβRII). Binding of TGF-β to TβRII induces the formation of a complex between TβRII and TβRI, leading to the activation of downstream signals [33]. The observations that Dlg5 depletion enhanced JNK and p38 activation, both of which are stimulated by TGF-β, and that overexpression of Dlg5 inhibited TGF-β induced-EMT raise the possibility that Dlg5-mediated regulation involves TGF-β receptor-dependent signaling. TGF-β receptor-mediated signaling can be stimulated by an autocrine mechanism. However, no increase in the secretion of latent or activated TGF-β into the medium was detected by ELISA assay (data not shown).

**Figure 6:**
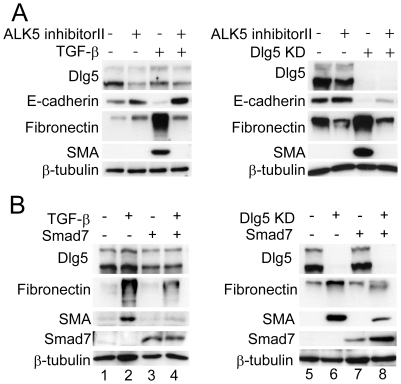
Inhibition of the TGF-β receptor suppresses the expression of mesenchymal marker proteins in Dlg5-depleted cells. **A:** LLc-PK1 cells were transfected with Dlg5 siRNA (KD) or control siRNA and stimulated with or without 4 ng/ml TGF-β. Two days after the addition of 2 µM ALK5 inhibitor II (TGF-β receptor inhibitor), cells were lysed and immunoblotted using the indicated antibodies. **B:** LLc-PK1 cells were transfected with a Smad7 expression plasmid or control plasmid and incubated for six hours. The cells were then treated with or without TGF-β (left panel) or transfected with Dlg5 siRNA or control siRNA (right panel). Two days after further incubation, cells were lysed and the expression of the indicated proteins was investigated by immunoblotting. Expression of β-tubulin was detected as a loading control. The results are representative of at least three independent experiments. Both pharmacological and physiological inhibition of TGF-β receptor suppressed the expression of SMA and fibronectin induced in Dlg5-depleted cells.

To examine whether TGF-β receptor is involved in EMT induced by Dlg5-depletion, a chemical inhibitor of the TGF-β receptor, ALK5 inhibitor II, was employed. LLc-PK1 cells transfected with Dlg5 siRNA or stimulated with TGF-β were treated with ALK5 inhibitor II. Inhibition of the TGF-β receptor by ALK5 inhibitor II almost completely suppressed the increase in fibronectin and SMA expression observed in Dlg5-depleted cells and in TGF-β-stimulated cells (Fig. 6A). ALK5 inhibitor II also rescued E-cadherin expression in Dlg5-depleted cells. The partial rescue of E-cadherin expression may indicate that other signals also contribute to the decrease in E-cadherin expression induced by Dlg5 depletion. Together, these results suggest that TGF-β receptor-mediated signaling is involved in EMT induced by Dlg5 depletion.

Smad7 is a well-known physiological inhibitor of TGF-β receptor signaling by promoting the degradation of the TGF-β receptor and by inhibiting the phosphorylation of Smad2/3 [34], [35], [36]. When LLc-PK1 cells were transfected with a plasmid expressing Smad7, the TGF-β-induced increase in SMA and fibronectin expression was suppressed as expected (Fig. 6B, lanes 2 and 4). Exogenous expression of Smad7 also suppressed the increases in fibronectin and SMA expression observed in Dlg5-depleted LLc-PK1 cells (lanes 6 and 8). Together, these results using chemical and physiological inhibitors indicate a role of Dlg5 in TGF-β receptor-mediated signaling, which is subsequently involved in the regulation of EMT.

## Discussion

Dlg5 is a member of the MAGUK adaptor family of proteins that localizes to cell-cell adhesion sites. Dlg5 has been reported to be one of the susceptibility genes for Crohn's disease [5], [37]. However, the physiological function of Dlg5 in cells is poorly understood. Here, we show that Dlg5 depletion changes the epithelial cell morphology of LLc-PK1 cells to an elongated fibroblast-like morphology. Concomitantly, Dlg5 depletion resulted in a decrease in E-cadherin expression and an increase in the expression of the mesenchymal markers, SMA and fibronectin. Immunostaining analysis further confirmed the disappearance of E-cadherin from cell-cell adhesion sites and the disruption of adherens junctions, suggesting a critical role of Dlg5 in the regulation of the EMT and the maintenance of the integrity of epithelial cells.

Dlg5 knockout (−/−) mice have penetrant hydrocephalus and kidney cysts [13]. Loss of apical-basolateral polarity in epithelial cells of the collecting ducts has been proposed to be a causal factor in kidney cysts. Because apical-basolateral polarity requires proper cell-cell adhesions and epithelial cell morphology, our finding that decreased Dlg5 expression disrupts cell-cell adhesions and induces EMT in renal epithelial LLc-PK1 cells may explain the mechanism behind the loss of apical-basolateral polarity in renal epithelial cells in Dlg5 (−/−) mice, although LLc-PK1 cells are derived from renal proximal tubules but not from collecting ducts. The high level of Dlg5 expression observed in human proximal tubule epithelial cells by immunohistochemistry suggests a significant role of Dlg5 in these cells (unpublished data). It is of interest that cysts found in the kidneys of Dlg5 knockout mice have a fibrous surface [13], because fibroblasts derived from tubular epithelial cells *via* EMT are a major source for the secretion and deposition of excess extracellular matrix in renal fibrosis [38]. Together, these observations suggest a pivotal role of Dlg5 in regulating EMT in renal epithelial cells.

TGF-β receptor signaling has a variety of functions, including the induction of growth arrest, cell death, and cell migration, in addition to the regulation of EMT. Tumor metastasis also involves the aberrant activation of TGF-β receptor signaling. We show that TGF-β receptor signaling mediates EMT induced by Dlg5 depletion. First, Dlg5 depletion activated JNK and p38 MAP kinases, which are both activated by TGF-β. Inhibition of these kinases rescued the expression of E-cadherin and suppressed the increases in SMA and fibronectin expression. Secondly, EMT induced by Dlg5 depletion was inhibited by either pharmacological or physiological inhibitors of the TGF-β receptor. A recent study [39] showing that Dlg5 inhibits cell migration and the expression of fibronectin and PAI-1, both of which can be regulated by TGF-β, in mammary epithelial cells also supports our findings. Interestingly, expression of Dlg5 in pancreatic cancers correlates with metastasis to the lymph nodes [40]. Reduction of Dlg5 expression significantly inhibited the proliferation of pancreatic cancer cell lines [41]. Although TGF-β signaling was not examined in these studies, our finding that Dlg5 can regulate TGF-β signaling is consistent with these reports. Furthermore, inflammatory bowel diseases, including Crohn's disease, involve dysfunction of TGF-β signaling [42], [43], [44], [45]. Thus, Dlg5 may also affect the pathology of Crohn's disease *via* regulation of TGF-β signaling. Future studies are necessary to examine this possibility.

Here, we demonstrate that Dlg5 depletion results in a decrease in E-cadherin protein and mRNA expression in LLc-PK1 cells. This decrease is dependent on TGF-β receptor signaling. In contrast, delivery of N-cadherin to the plasma membrane has been reported to be impaired in Dlg5 (−/−) fibroblasts, leading to a decrease in cell-surface but not total expression of N-cadherin [13]. The association of Dlg5 with β-catenin and the t-SNARE complex has been suggested to be involved in its delivery to the cell surface [13]. In LLc-PK1 cells, however, the ratio of cell surface to total expression of E-cadherin and N-cadherin was not affected by Dlg5 depletion (data not shown). This observation is consistent with that observed in MCF10A cells [39]. Furthermore, knockdown of β-catenin expression did not affect the EMT induced by Dlg5 depletion. Therefore, these observations suggest that different mechanisms are used to regulate the cell surface expression of cadherins in fibroblasts and in LLc-PK1 or MCF10A epithelial cells.

TGF-β triggers Smad signaling pathways and non-Smad MAPK signals [32], [46]. Our study showed that Dlg5 depletion activated non-Smad signals, JNK and p38, but not Smad2 phosphorylation. Dlg5 depletion also increased mRNA expression of *Snail*, an important regulator of EMT. JNK and p38 as well as Smad signals induce the expression of Snail in renal epithelial cells and other cell types [47], [48], [49], [50]. Thus, *Snail* may mediate the effect of p38 and JNK on EMT induced by Dlg5 depletion. TRAF6 has recently been reported to associate with TβR and induce Smad-independent activation of p38 and JNK but not Smad signaling. TAK1 also interacts with TβR and stimulates p38 and JNK activation [51], [52], [53]. The distribution of TβR to lipid rafts and non-rafts also plays a role in Smad and non-Smad signaling [54]. We examined TAK1 activation and the distribution of TβR to lipid rafts, but we have not yet obtained any evidence suggesting that Dlg5 affects these events, although interaction between exogenously expressed Dlg5 and TβRI was observed (unpublished data). Dlg5 may affect another mechanism that regulates non-Smad signaling but not Smad signaling.

In summary, we found that TGF-β-induced EMT suppresses Dlg5 expression in LLc-PK1 cells. Depletion of Dlg5 expression by knockdown promoted the expression of the mesenchymal marker proteins, fibronectin and SMA, and suppressed the expression of E-cadherin. JNK and p38 activation, which are stimulated by TGF-β, were enhanced by Dlg5 depletion. Furthermore, induction of fibronectin and SMA expression by Dlg5 depletion was dependent on TGF-β receptor-mediated signaling. These observations suggest that Dlg5 is involved in the regulation of TGF-β receptor-dependent signaling and EMT in LLc-PK1 cells.

## Supporting Information

Figure S1 **The expression of Dlg5 and vinexin α/β after TGF-β stimulation.** A: LLc-PK1 cells were incubated with 4 ng/ml of TGF-β for two days. Total RNA was isolated from the cells and *Dlg5* mRNA was quantitated by real-time PCR. The values represent the mean ± S.E. of relative mRNA amounts from three independent experiments. B: LLc-PK1 cells were incubated with 4 ng/ml of TGF-β for three days. The expression of vinexin α and β was determined by immunoblotting. As a loading control, vinculin expression was detected.
(TIF)

Figure S2 ***E-cadherin*** **mRNA in Dlg5-knockdown cells.** Control siRNA (GFP siRNA or control siRNA1 (unrelated)) or *Dlg5* siRNA (#1 or #2) was transfected into LLc-PK1 cells. Two days after transfection, total RNA was isolated from the cells and *E-cadherin* mRNA was quantitated by real-time PCR. The values represent the mean ± S.E. of relative mRNA amounts from three independent experiments.
(TIF)

Figure S3 **Effect of siDlg5#2 on expression of SMA and fibronectin.** LLc-PK1 cells were treated with 4 ng/ml TGF-β or transfected with control siRNA or Dlg5 siRNA (#1, #2) followed by incubation for three days. Cells were lysed and protein expression detected by immunoblotting using the indicated antibodies. β-tubulin expression was examined as a loading control. A line was inserted to indicate a vertically spliced lane; however, all samples were loaded on the same gel.
(TIF)

Figure S4 **Effect of depletion of Dlg5 expression in PC3 cells.** shRNA plasmid for Dlg5 or control plasmid were introduced into PC3 cells using lentivirus. Transfected cells were selected by the incubation with 1.0 µg/ml puromycin. Lysates were prepared from cells stably transfected with shRNA for Dlg5 or control plasmid. The cell lysates were immunoblotted using the indicated antibodies. β-tubulin expression was detected as a loading control. Depletion of Dlg5 expression in PC3 cells induced the increase in fibronectin expression and the decrease in E-cadherin expression.
(TIF)

Figure S5 **Effect of β-catenin knockdown.** Dlg5 siRNA and β-catenin siRNA were transfected into LLc-PK1 cells with or without stimulation with 4 ng/ml TGF-β. After three days of incubation, cells were lysed and protein expression detected by immunoblotting using the indicated antibodies.
(TIF)

Figure S6 ***Snail*** **mRNA in TGF-β treated and Dlg5-knockdown cells.** LLc-PK1 cells were transfected with control siRNA or *Dlg5* siRNA#1 (A) or treated with 4 ng/ml TGF-β (B). After two days of incubation, total RNA was isolated from the cells and *Snail* mRNA was quantitated by real-time PCR. The values represent the mean ± S.E. of relative mRNA amounts from three independent experiments.
(TIF)
