# Supplementary figures and images for: Role of Dlg5/lp-dlg, a Membrane-Associated Guanylate Kinase Family Protein, in Epithelial-Mesenchymal Transition in LLc-PK1 Renal Epithelial Cells
Source: PLoS One. 2012 Apr 23;7(4):e35519. doi: 10.1371/journal.pone.0035519 (PMC3335148; doi:10.1371/journal.pone.0035519)

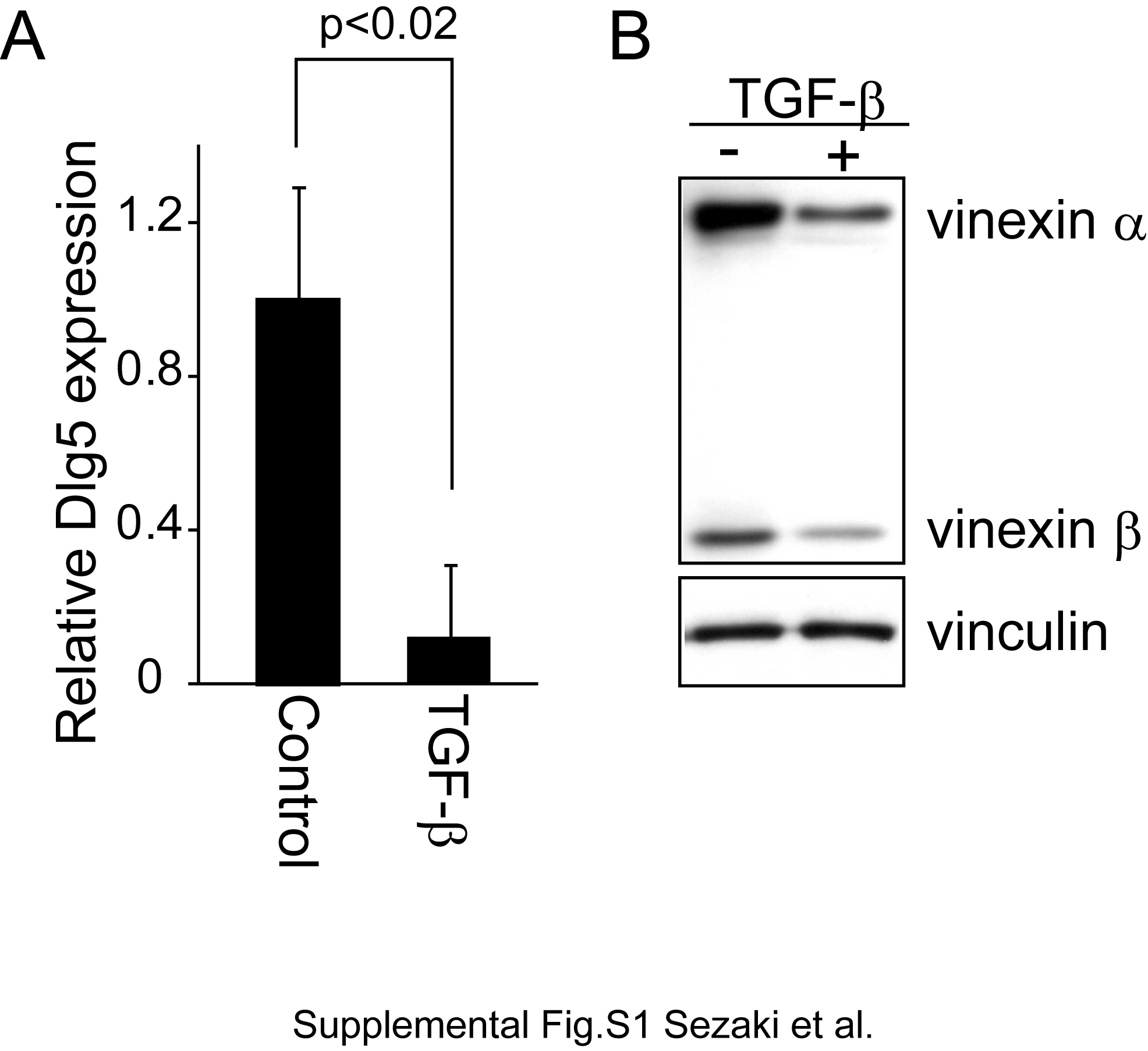

Supplement: Figure S1 — The expression of Dlg5 and vinexin α/β after TGF-β stimulation. A: LLc-PK1 cells were incubated with 4 ng/ml of TGF-β for two days. Total RNA was isolated from the cells and Dlg5 mRNA was quantitated by real-time PCR. The values represent the mean ± S.E. of relative mRNA amounts from three independent experiments. B: LLc-PK1 cells were incubated with 4 ng/ml of TGF-β for three days. The expression of vinexin α and β was determined by immunoblotting. As a loading control, vinculin expression was detected. (TIF) [file pone.0035519.s001.tif]

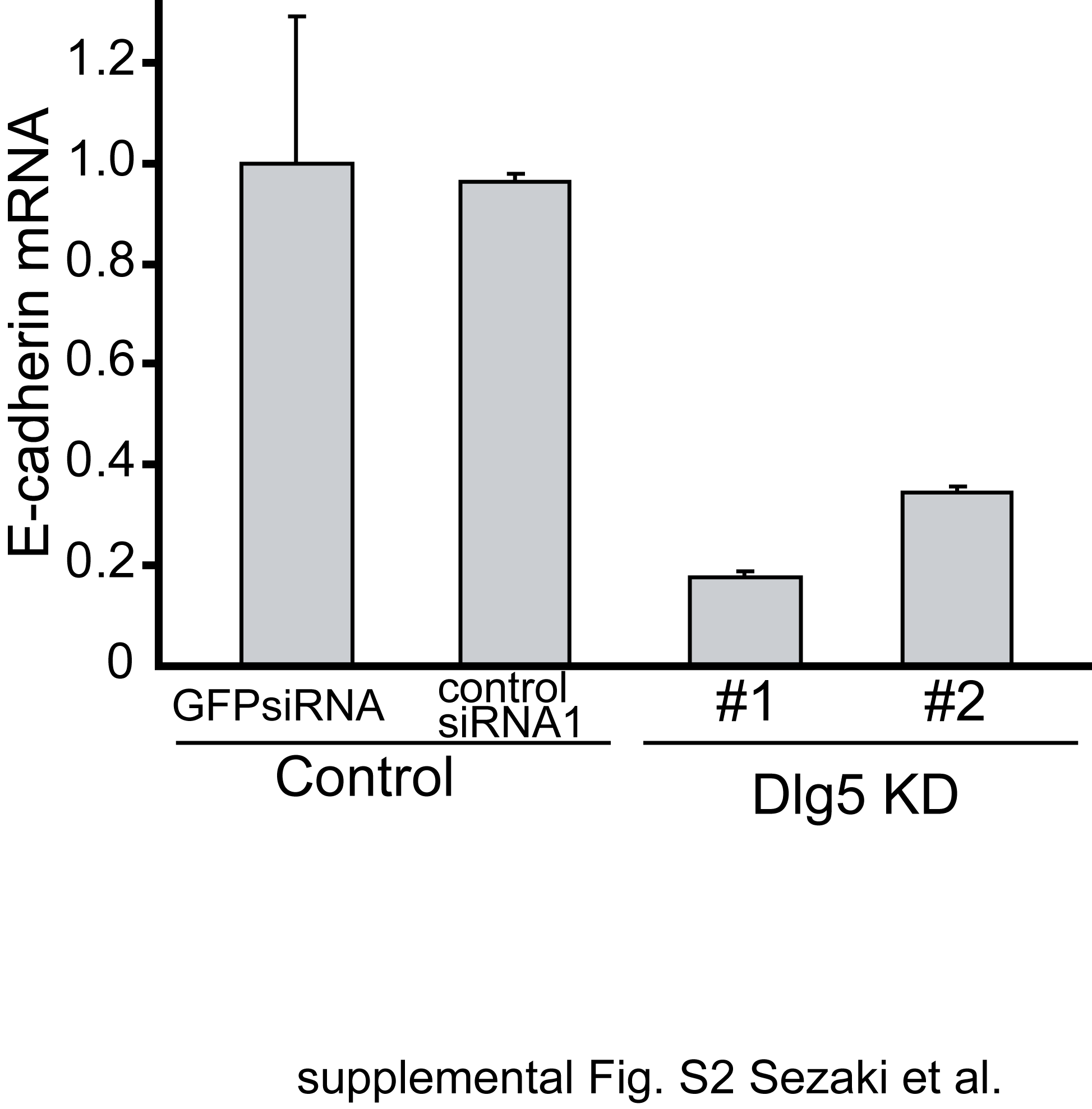

Supplement: Figure S2 — E-cadherin mRNA in Dlg5-knockdown cells. Control siRNA (GFP siRNA or control siRNA1 (unrelated)) or Dlg5 siRNA (#1 or #2) was transfected into LLc-PK1 cells. Two days after transfection, total RNA was isolated from the cells and E-cadherin mRNA was quantitated by real-time PCR. The values represent the mean ± S.E. of relative mRNA amounts from three independent experiments. (TIF) [file pone.0035519.s002.tif]

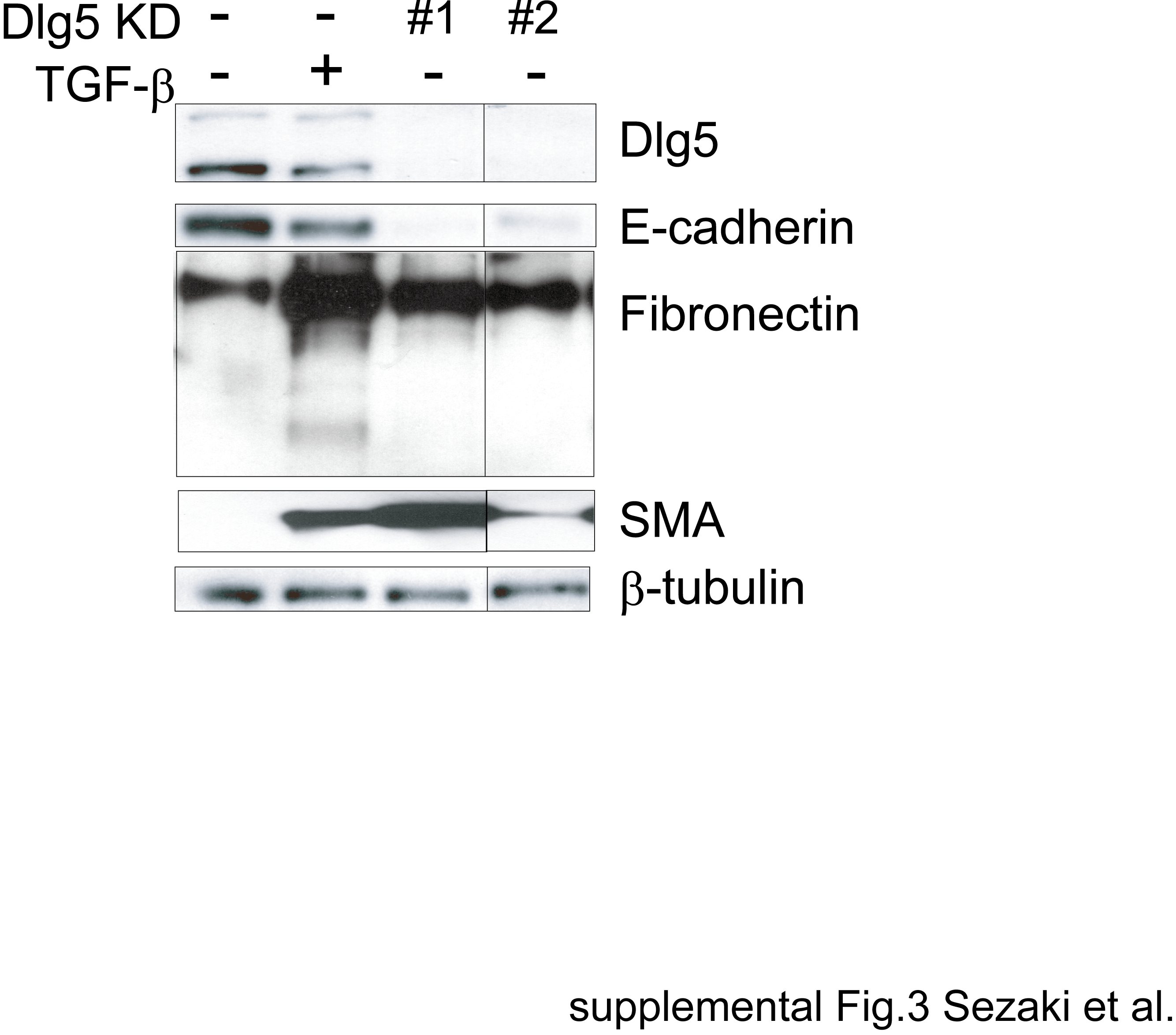

Supplement: Figure S3 — Effect of siDlg5#2 on expression of SMA and fibronectin. LLc-PK1 cells were treated with 4 ng/ml TGF-β or transfected with control siRNA or Dlg5 siRNA (#1, #2) followed by incubation for three days. Cells were lysed and protein expression detected by immunoblotting using the indicated antibodies. β-tubulin expression was examined as a loading control. A line was inserted to indicate a vertically spliced lane; however, all samples were loaded on the same gel. (TIF) [file pone.0035519.s003.tif]

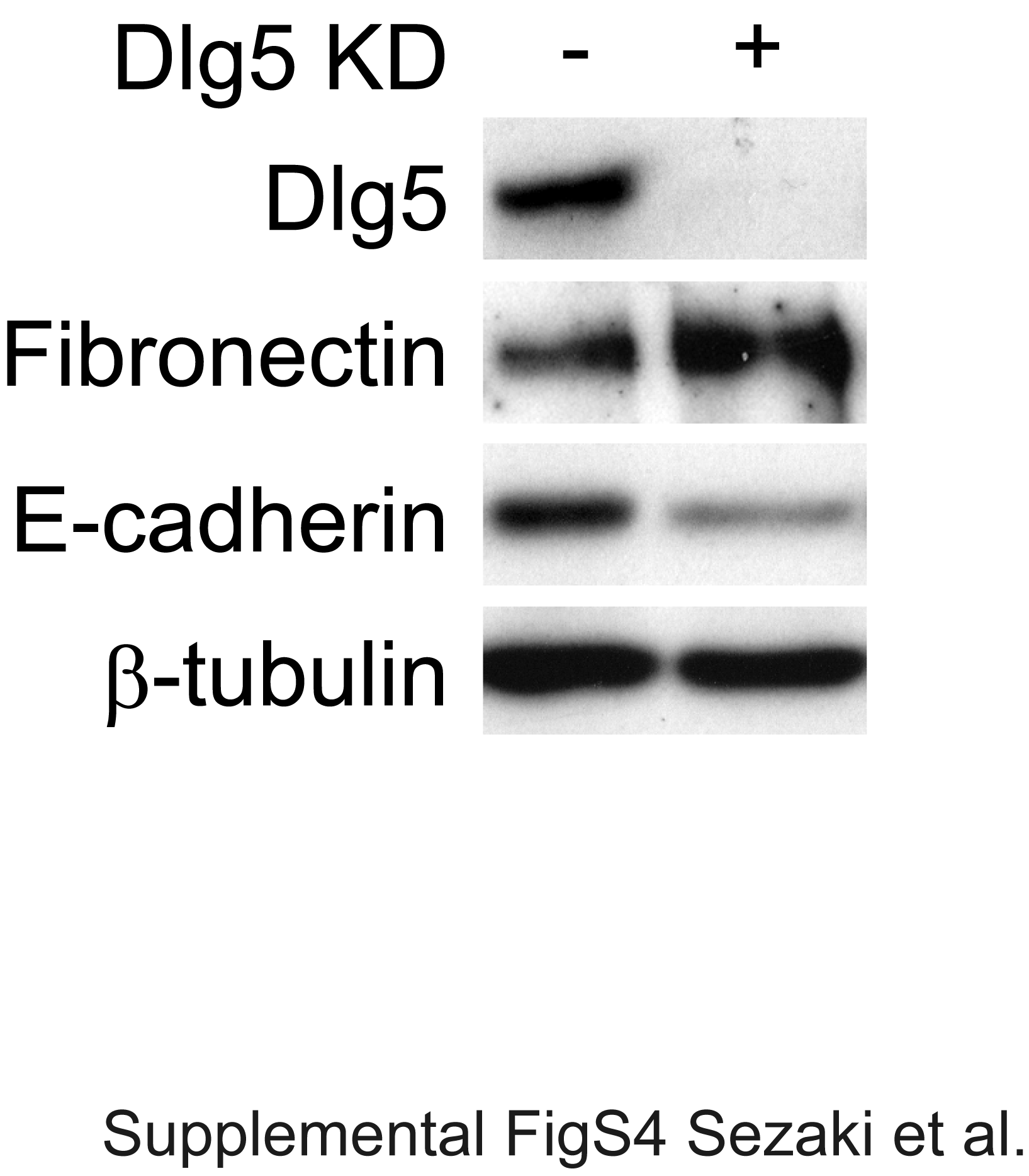

Supplement: Figure S4 — Effect of depletion of Dlg5 expression in PC3 cells. shRNA plasmid for Dlg5 or control plasmid were introduced into PC3 cells using lentivirus. Transfected cells were selected by the incubation with 1.0 µg/ml puromycin. Lysates were prepared from cells stably transfected with shRNA for Dlg5 or control plasmid. The cell lysates were immunoblotted using the indicated antibodies. β-tubulin expression was detected as a loading control. Depletion of Dlg5 expression in PC3 cells induced the increase in fibronectin expression and the decrease in E-cadherin expression. (TIF) [file pone.0035519.s004.tif]

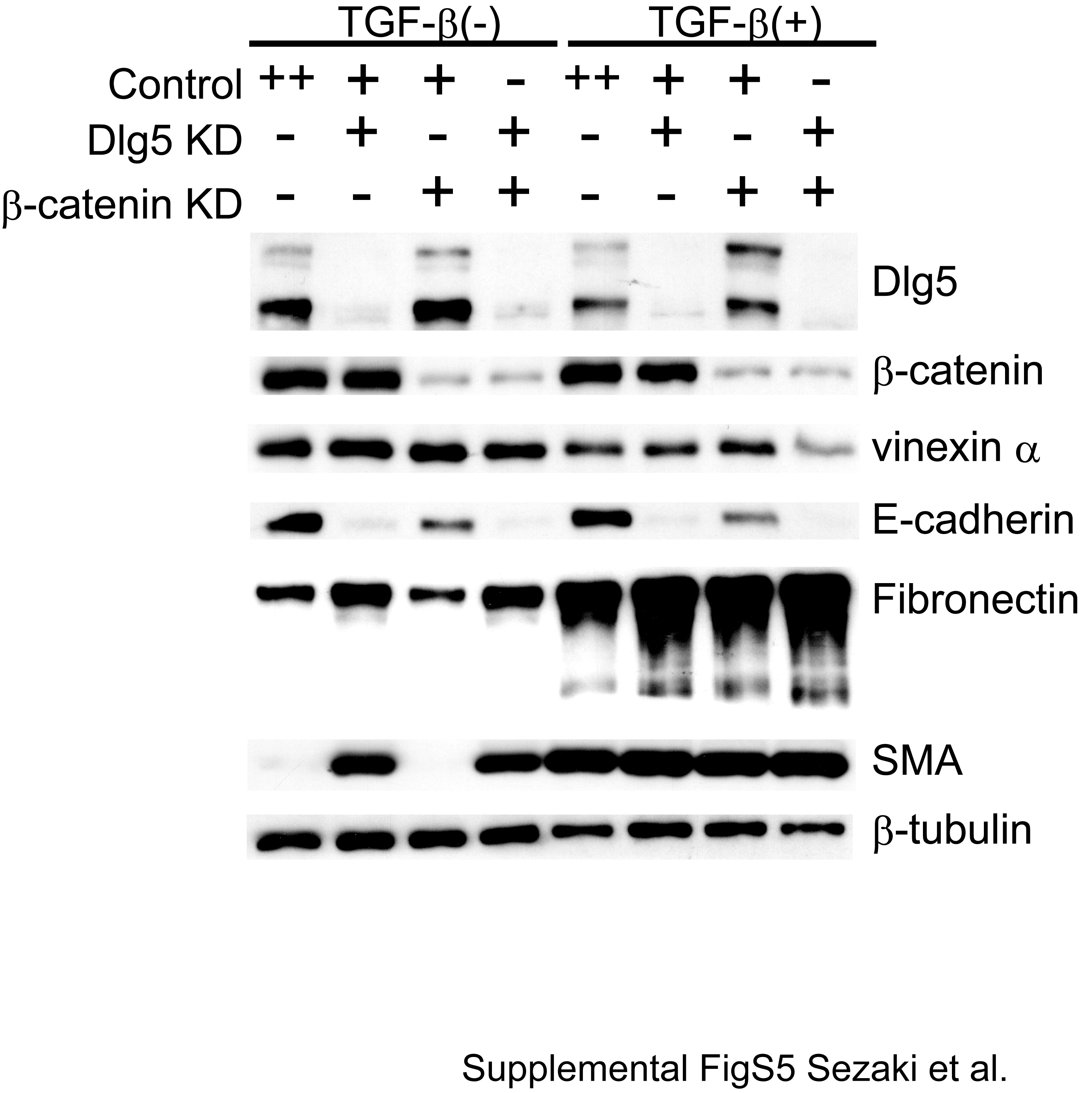

Supplement: Figure S5 — Effect of β-catenin knockdown. Dlg5 siRNA and β-catenin siRNA were transfected into LLc-PK1 cells with or without stimulation with 4 ng/ml TGF-β. After three days of incubation, cells were lysed and protein expression detected by immunoblotting using the indicated antibodies. (TIF) [file pone.0035519.s005.tif]

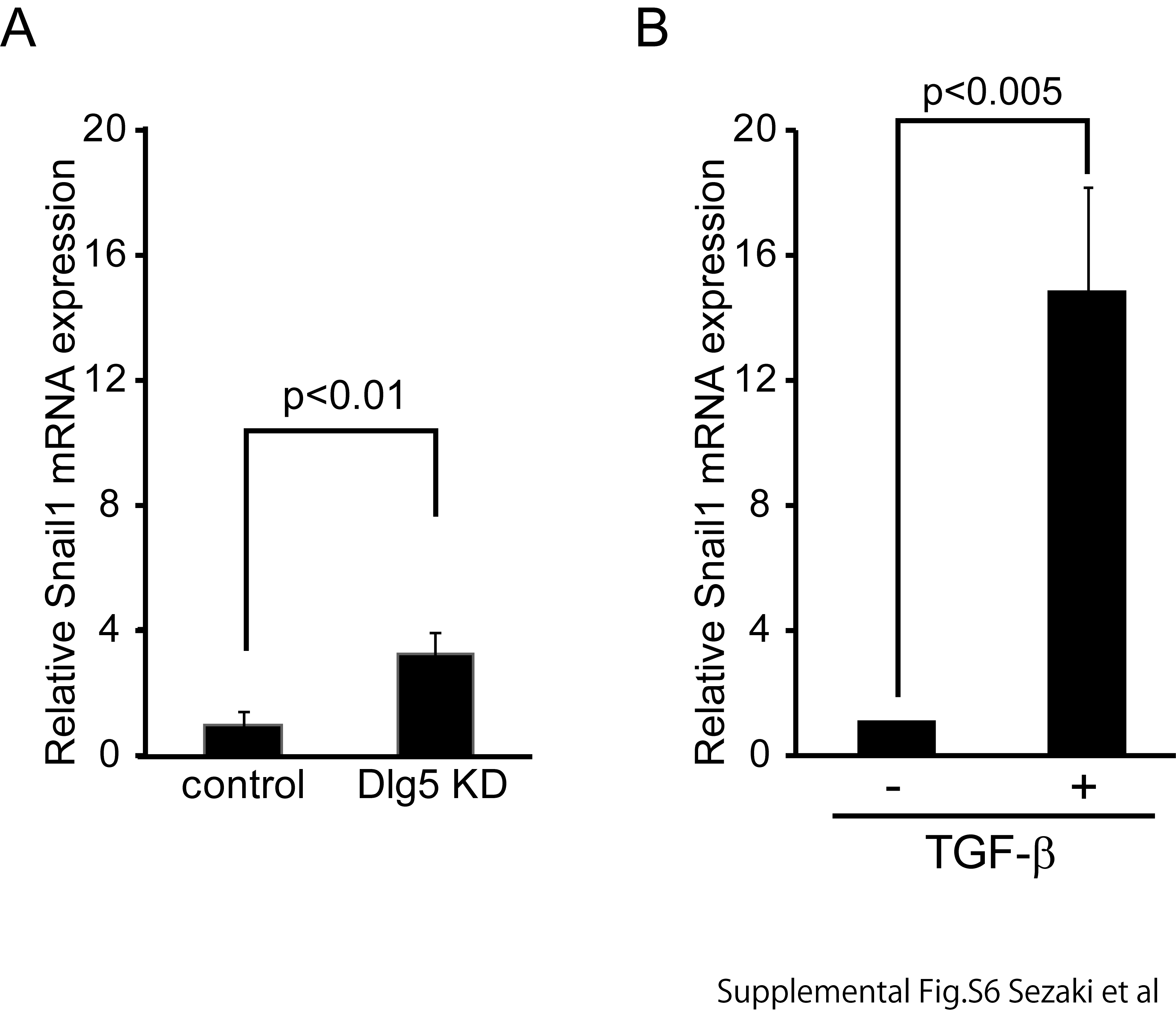

Supplement: Figure S6 — Snail mRNA in TGF-β treated and Dlg5-knockdown cells. LLc-PK1 cells were transfected with control siRNA or Dlg5 siRNA#1 (A) or treated with 4 ng/ml TGF-β (B). After two days of incubation, total RNA was isolated from the cells and Snail mRNA was quantitated by real-time PCR. The values represent the mean ± S.E. of relative mRNA amounts from three independent experiments. (TIF) [file pone.0035519.s006.tif]
